# Man and His Enemies

**Published:** 1889-05-25

**Authors:** 


### The Doctor's Pulpit.

### MAN AND HIS ENEMIES.

### "A Faint Heart."

"A faint heart never won a fair lady." Is any single proverb of Solomon's so widely known as this? It is said that Leicester, whose ambition soared as high as the hand of the Maiden Queen, once wrote on a window-pane, where Elizabeth was sure to see it, the following couplet:

"Fain would I climb,
Yet fear I to fall."

As was to be expected, the Queen did see it, and immediately wrote underneath:

"If thy heart fail thee,
Climb not at all."

The difference between a valiant and a cowardly heart is immeasurable by human methods of computation. The heroic man approaches the divine: the coward is baser than the meanest dog. And yet very few men are by nature boldly, stedfastly, unflinchingly brave. It is reported of a gallant and distinguished officer that he never led his troops into an engagement without turning deadly pale, with what he himself and those who knew him could only regard as fear. A certain raw subaltern once noticed this, and, without thinking, blurted out, "Why, colonel, you're afraid!" "Yes," said the colonel, "and if you were half as frightened as I am you would run away!" That colonel not only never ran away, but was one of the bravest officers in action the British army has ever known.

The "heart" with which a man approaches life and its work is so fundamental a matter that it is well worth many a two or three minutes' consideration. Most men in early life have the ambition to do great things; and if there were any conjuring trick which, when once learnt, would ensure the doing of them, the master of that trick would never lack pupils. A good many have not merely the ambition to "cut a figure," but they have also the courage to make a start. School and college competitions always bring up a large number of starters, and not a few hold on even to the end of the last college contest. But the race of life thins them out. So soon as a man is shaken free from the supports and restraints of pupillage, and, stripping to his work, starts off among his fellows on his own account, then the spectators begin to take his measure and to make up their minds where he is to be when the race is over.

What has he entered for? Does he know? Has he made up his mind? Or has he plunged into the competition not knowing what he would be at, or without once thinking what are the conditions of the race? Many men, perhaps most, enter for a short heat and for an immediate prize. The far-seeing and the large-minded enter for a life struggle. It is only to these last that any permanently worthy prize is likely to come. Immediate success is generally like all other quick-grown fruit; it does not "keep" very long. No doubt many a senior wrangler thinks his life victory won when he sees his name in front of those of all his competitors. Of course, the real strife has not yet commenced; and it often happens that even senior wranglers make name and fame in some subject far removed from mathematics, whilst many, perhaps the majority of them, never make either name or fame at all.

Whatever be a man's equipment in the way of "heart," it is quite certain that the work to be done and the war to be waged in life demand substantial and steady courage. He who has not that is already beaten. Courage is distinctly of two kinds. One is a sort of natural daredevilness. Certain persons seem never to know fear. That kind of courage, which the unthinking rate very high, and which indeed, many people regard as the only real courage, is of an altogether inferior quality as compared with the true gold. The highest, and perhaps the only genuine courage, is that which is born of reasoning, forethought, and the deliberate making up of the mind that a certain course is to be pursued at any and every cost. Some, even among bold people, never display courage of this kind at all. The writer knows more than one eminent and successful person who can be turned from courses they have marked out for themselves, courses which are both right and wise, by quite lower and inferior considerations if sufficient pressure be brought to bea upon them. The highest prizes of life are not for these. Posterity will never place them in the front rank of its mighty. The eternal records will put no "well done" after their names.

Such courage as is here called the highest and truest depends little upon a man's natural "stoutness of heart." It is quite possible indeed for many who have no "natural stoutness of heart" at all, but, on the contrary, a decided "natural shrinking," to display it in a very marked degree. The feeblest human creature can understand what is right, and can make up his mind to do it. The mind once made up, all that is needed is to stand firm; and then no power on earth can move a rock thus fixed. That is the kind of courage to front modern life with. No enemy of man can beat it down or root it up. It is of the very essence of mental and moral sanitation. Physical evils may, and probably will, beat against it; mental and moral evils most certainly will. To out ward appearance failure, complete and permanent, may place its foot on the neck of a man and lock him fast in its prison. But no failure ever conquered one who did not himself first cry "defeat." The nature of man is such, and has been such from the beginning of the world, that destiny itself cannot break him if he remain firm in mind.

This kind of courage is eminently needed in such a complicated civilisation as ours. The workman needs it as much as the statesman, and the woman as much as the man. Science is a powerful solvent, and the student often finds in these latter days that what he considered the solidest foundations have gradually melted away beneath his feet, and that he is threatened with engulfment if he cannot either swim or find new rocks to stand upon. A "faint heart" under these circumstances is a miserable foundation. Lord Beaconsfield prophesied failure for one of his characters because "he had no root," meaning thereby that he had no money and no estate. If the only permanent root a man can have be money or estate, then woe to the world: A root more permanent than either money, land, or reputation is the root of intelligent courage. He who has that, and is resolved to retain it, has an impregnable defence against all his enemies.

